# Inhaled salmeterol and/or fluticasone alters structure/function in a murine model of allergic airways disease

**DOI:** 10.1186/1465-9921-11-22

**Published:** 2010-02-24

**Authors:** Erik P Riesenfeld, Michael J Sullivan, John A Thompson-Figueroa, Hans C Haverkamp, Lennart K Lundblad, Jason HT Bates, Charles G Irvin

**Affiliations:** 1 Vermont Lung Center, University of Vermont, Burlington, Vermont, USA

## Abstract

**Background:**

The relationship between airway structural changes (remodeling) and airways hyperresponsiveness (AHR) is unclear. Asthma guidelines suggest treating persistent asthma with inhaled corticosteroids and long acting β-agonists (LABA). We examined the link between physiological function and structural changes following treatment fluticasone and salmeterol separately or in combination in a mouse model of allergic asthma.

**Methods:**

BALB/c mice were sensitized to intraperitoneal ovalbumin (OVA) followed by six daily inhalation exposures. Treatments included 9 daily nebulized administrations of fluticasone alone (6 mg/ml), salmeterol (3 mg/ml), or the combination fluticasone and salmeterol. Lung impedance was measured following methacholine inhalation challenge. Airway inflammation, epithelial injury, mucus containing cells, and collagen content were assessed 48 hours after OVA challenge. Lungs were imaged using micro-CT.

**Results and Discussion:**

Treatment of allergic airways disease with fluticasone alone or in combination with salmeterol reduced AHR to approximately naüve levels while salmeterol alone increased elastance by 39% compared to control. Fluticasone alone and fluticasone in combination with salmeterol both reduced inflammation to near naive levels. Mucin containing cells were also reduced with fluticasone and fluticasone in combination with salmeterol.

**Conclusions:**

Fluticasone alone and in combination with salmeterol reduces airway inflammation and remodeling, but salmeterol alone worsens AHR: and these functional changes are consistent with the concomitant changes in mucus metaplasia.

## Background

There is a variety of pathological changes that are therapeutic targets in asthma [1]. Principal among these is periodic or persistent inflammation, which is the cardinal feature of allergic asthma that presumably leads to the persistent structural changes known as remodeling. Remodeling includes a spectrum of alterations including collagen deposition, epithelial thickening, goblet cell hyperplasia and smooth muscle thickening. The overall functional consequences of airway remodeling remain uncertain [2], but the consequences are generally cast as detrimental. The propensity for the distal airways of asthmatics to become plugged with mucus is a well-known hallmark of fatal asthma [3]. Mucus also likely plays an important role in the distal airway closure that underlies the AHR of allergically inflamed mice [4-6]. Mitigation of the inflammation induced remodeling may therefore, be a key goal in asthma treatment.

Clinical guidelines call for asthma treatment with inhaled corticosteroids (ICS) and long acting β-agonists (LABA) for moderate and severe persistent asthma [7]. The combination of LABA and ICS is apparently more effective than simply doubling the dose of ICS [8]; however, the precise mechanism of the effect of the combined agents remains uncertain [9]. Despite the benefit of combination therapy, clinical trials have found adverse events associated with LABA used as monotherapy, leading the US FDA to institute "boxed warnings" related to LABA use [10-12]. The issue is further complicated by the results of a recent clinical trial suggesting that regular treatment with short acting bronchodilators might also be detrimental, even when used in combination with ICS [13].

The multiplicity of sites of action that ICS have in the inflammatory cascade explains why they are currently the most efficacious therapy for asthma [7,14]. However, it has been suggested that LABAs also have anti-inflammatory properties [9,15-17] in addition to being able to relax airway smooth muscle. With this combination of benefits, the finding that LABA use is associated with adverse outcomes would seem to be puzzling. On the other hand, studies of the anti-inflammatory properties of LABA have so far focused primarily on epithelial permeability and cellular accumulation in the lungs [17]. This is a limited spectrum of action compared to that attributed to ICS. It is therefore possible that the detrimental consequences of LABA use arise because other aspects of the inflammatory response are increased such as airway wall thickening and mucus hyper-secretion.

Accordingly, we hypothesized that LABA treatment would upregulate components of the inflammatory or "remodelling" response that exacerbate airway closure, and that this is prevented by concomitant use of ICS. To address this hypothesis, we focused on how airway hyperresponsiveness in allergically inflamed mice is modulated by treatment with an inhaled LABA (salmeterol), or ICS (fluticasone), or the combination of the two. We related these physiological outcomes to measures of airway and parenchymal remodeling based on histological indices and micro-CT imaging.

## Methods

Experiments were approved by the Institutional Animal Care and Use Committee of the University of Vermont.

### Animals and the OVA Allergic Airways Disease model

**Figure 1:**
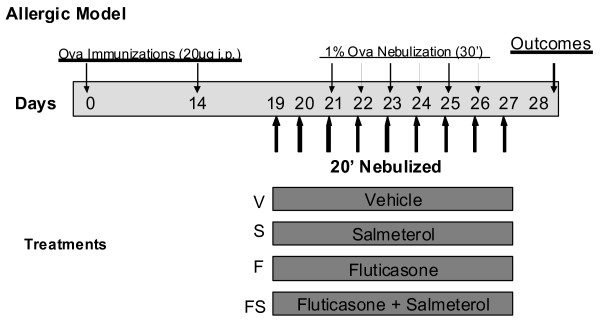
**Experimental Study Design Scheme**. BALB/c mice were immunized intraperitoneally with 20 micrograms of Ovalbumin (OVA) on days 0 and 14. OVA was then nebulized daily as a challenge on days 21 to 26. Different groups of mice were treated with 20 minute nebulizations of vehicle, fluticasone 6,000 micrograms per ml, salmeterol 3,000 micrograms per ml, or the combination of fluticasone and salmeterol. These were dosed once daily from days 19 to 27 (9 doses).

Female BALB/c mice (age 6-12 weeks with n = 6-8 per group from Jackson Laboratories, Bar Harbor, ME) were sensitized to ovalbumin (OVA) (Sigma-Aldrich St. Louis, MO) with alum adjuvant (aluminum hydroxide) (Pierce Chemical, Rockford, IL) as previously described [18]. The experimental study design scheme is shown in Figure 1. Because of technical limitations imposed by the protocol for computed tomography (CT) imaging, half of each group were subjected to CT imaging whereas the other half had BAL and histology performed. Mice received intraperitoneal OVA and alum (days 0 and 14) followed by nebulized 1% OVA in sterile phosphate buffered saline on days 21-26 (O group). A naïve (N) group served as a control. Nebulized treatments were given for 30 minutes in a compartmentalized exposure chamber using an attached Pari LC plus^® ^nebulizer with a Proneb^® ^Ultra II (PARI Innovative Manufacturers, Inc Midlothian, VA).

### Drug Treatments

The O group was sub-divided to receive the following nebulized treatments; vehicle control (V) (D-PBS/0.17% tween 80), fluticasone (F) (6 mg/ml), salmeterol (S) (3 mg/ml) or the combination of salmeterol (3 mg/ml) and fluticasone (FS) (6 mg/ml) (GlaxoSmithKline Middlesex, UK). Drugs were administered once a day for 20 minutes using the same nebulizer arrangement described above in the OVA model section on days 19-27 and data were collected on day 28 (24 hours after the last dose).

### Lung Mechanics

Mice were anesthetized with pentobarbital (90 mg/kg), tracheostomized and ventilated with room air at a rate of 200 breaths per minute with a tidal volume of 0.25 ml and positive end expiratory pressure of 3 cm H_2_O (flexiVent, Scireq, Montreal). Mice received 2 sighs limited to a pressure of 25 cm H_2_O. Following this, two baseline measurements of respiratory input impedance (*Z*_*rs*_) were made followed by nebulized methacholine challenges (saline control and methacholine at 3.125, 12.5, and 50 mg/ml). Methacholine was nebulized for 40 seconds with the inspiratory line of the ventilator connected through a nebulizer (Beetle-Neb Ultrasonic Nebulizer Drive Medical Design and Manufacturing Port Washington, NY) using a tidal volume of 0.8 ml with a rate adjusted to provide the same minute ventilation as the baseline ventilation.

Newtonian Resistance (*R*_*N*_), tissue resistance or damping (*G*), and elastance (*H*) were calculated by fitting the constant-phase model to respiratory impedance as described previously [19-22]. Mice were then euthanized followed by either a CT scan or a bronchoalveolar lavage (BAL) [4,23].

### Histology

Bronchoalveolar lavage (BAL) cell counts were recorded as previously described [24]. Lungs were inflation fixed with 10% formalin at 30 cm pressure and stained with Hematoxylin and Eosin (H+E), Sirius red (for collagen) [25], or fluorescent periodic acid Schiff (PAFS) to evaluate mucus containing cells as per Evans et al. [26]. PAFS staining was used due to its greater specificity with less background staining than the standard PAS stain. Immersion fixation was done with additional mice (2 from O and 2 from FS) so that the luminal space could be visualized without disruption caused by lavage or inflation.

### Morphometry

Semi-quantitative assessment of inflammation, collagen deposition and epithelial damage was performed by three masked readers. Epithelial thickness, collagen, and mucin containing cells were quantified using customized Image J software (see online supplement for a detailed description) [27]. Slides were viewed (Zeiss, Axioskop 2 plus, Göttingen, Germany) at 10 × or 20 ×. Scoring for inflammation and epithelial damage used a four point scale (0-least to 3-most). The epithelial damage score incorporated epithelial cell thickness and cell disruption. Collagen was determined quantitatively and semi-quantitatively from polarized Sirius Red stained slides (see additional file 1 for details). PAFS positive cells were recorded as a number of cells per micron of basement membrane. Epithelial thickness was measured as the area between the luminal cell membrane and the basement membrane (BM) divided by the BM length in microns.

### Computed Tomography

After euthanasia, mice the mouse trachea was tied off at 3 cm H_2_O and imaged at 80 kV and 450 mA for 80 min using a micro-CT scanner (eXplore, GE Medical systems) [4]. Images were converted into iso-surface renderings for visualization of the air-tissue interface. Thoracic gas volume (V_TG_) was determined by summing the fractions of air in each pixel as previously described by Lundblad et al. [4].

### Statistics

Statistics were calculated using Origin 7.5 (OriginLab Corp, Northampton, MA). ANOVA followed by Tukey-Kramer pairwise comparisons were used to compare treatment effects. Lung mechanics parameters were compared using a two way ANOVA followed by a means comparison using a Tukey test. Data are expressed as mean ± SE. Significance was taken as p < 0.05.

## Results

### Bronchoalveolar Lavage

**Figure 2:**
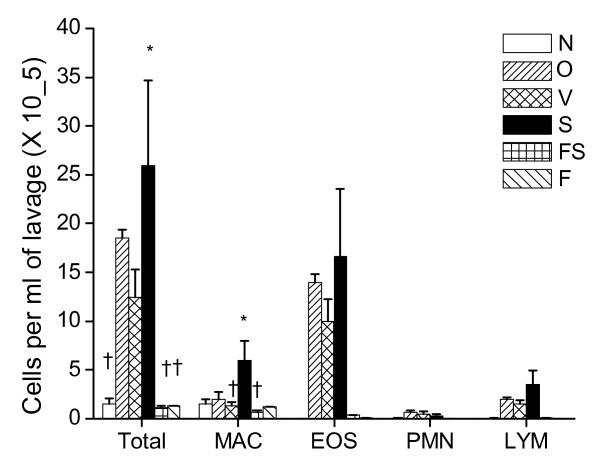
**BALF Cell Counts**. Total cells (Total), macrophages (MAC), eosinophils (EOS), neutrophils (PMN), and lymphocytes (LYM). Treatment groups; Naïve mice (N), Inhaled OVA (6 doses) (O), OVA with vehicle control (V), salmeterol (S), fluticasone (F), and the combination (fluticasone and salmeterol) (FS). Mean cells per ml of BAL fluid ± SE. * in Total cells p < 0.05 for S compared to. N, FS, and F. † in Total cells p < 0.05 for N, F and FS compared to S. * in MAC p < 0.05 for S compared to V and FS. † in MAC p < 0.05 for V and FS compared to S.

The BAL cellularity was greater in the O and V and S groups compared to N, F and FS. Variability in the cell counts limited the statistical significance with the conservative statistical test of an ANOVA with Tukey's Multiple Comparison Test (see Figure 2) (p < 0.01 for ANOVA). The greatest cellularity was seen in the S group but significance was noted only for S compared to N, F and FS for total cells. Cell counts were at naüve levels in both the F and FS treated groups. BALF fluid return ranged from 0.6-0.9 ml per mouse.

### Histology

**Figure 3:**
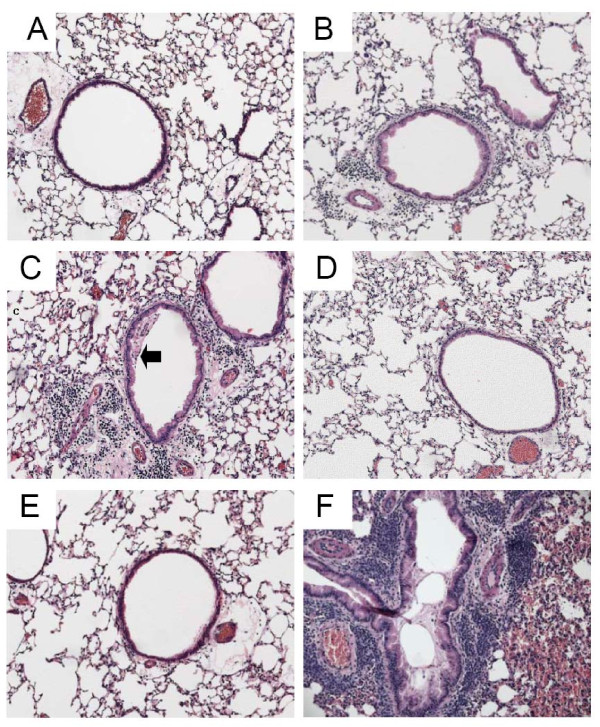
**Histology**. Representative* Hematoxylin and Eosin stained tissue sections taken with 10× objective. A) Naïve, B) OVA, C) Salmeterol (arrow indicates mucus adherent to wall), D) Fluticasone, E) Fluticasone and Salmeterol, F) immersion fixed lung from OVA challenged mouse demonstrating airway obstruction with mucus in bronchial lumen. *Representative figures were chosen using criteria described in the text.

**Figure 4:**
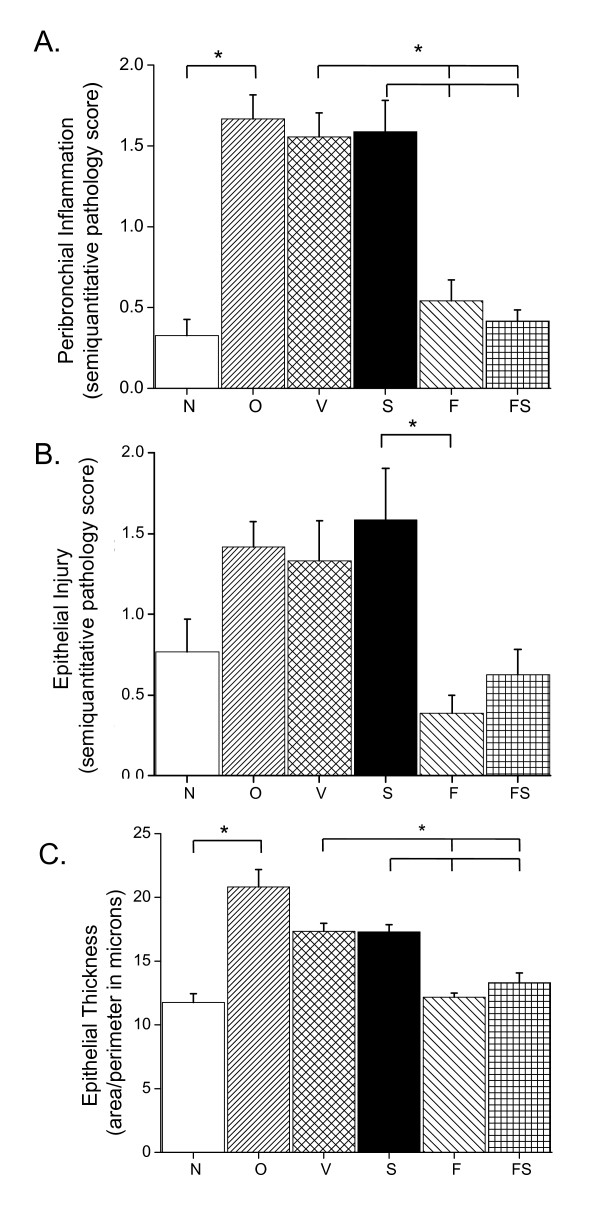
**Tissue Scores**. Peribronchial inflammation, epithelial thickening and injury. A) Semi-quantitative score for peribronchial inflammation. B) Semi-quantitative score for global epithelial damage. C) Quantitative epithelial thickness. Groups; naïve mice (N), OVA (O), and O mice with vehicle control (V), salmeterol (S), fluticasone (F), and a combination of fluticasone and salmeterol (FS). N = 4-6 mice in each group with 4 airways per mouse (averaged for each mouse/slide). Results expressed as mean ± SE. * p < 0.05.

Figure 3 presents representative photomicrographs from each group. These images have pathology scores similar to their respective group means shown in Figure 4. Sensitization and challenge with O caused a significant increase in peribronchial inflammation in the O group compared to the N group. Fluticasone, either alone (F) or in combination with salmeterol (FS), dramatically reduced peribronchial inflammation to N group levels (see Figure 3, panels D and E). There was no evidence of reduced inflammation in the S group in which mucus frequently adhered to the airway wall as depicted in Figure 3, panel C. In comparing the scores of the three readers for inflammation, an Intraclass Correlation Coefficient (ICC) was calculated to be 0.861. P values for Pearson correlations were all < 0.0017. Mucus plug formation and abundant peribronchial inflammation were seen in the OVA treated lungs that were immersion fixed (see Figure 3 panel F).

Scores of epithelial injury and thickness are also shown in Figure 4. OVA caused an increase in epithelial thickening that was reduced to naive levels with nebulized fluticasone. Epithelial damage and thickening were greatest in the O, V and S groups. The thickness of the epithelium was less variable than the global pathology score, and there was no difference attributable to airway size in either endpoint (data not shown).

There was no significant change in peribronchial airway collagen deposition assessed by Sirius red staining at the 28 day time point (data not shown).

### Physiology

**Figure 5:**
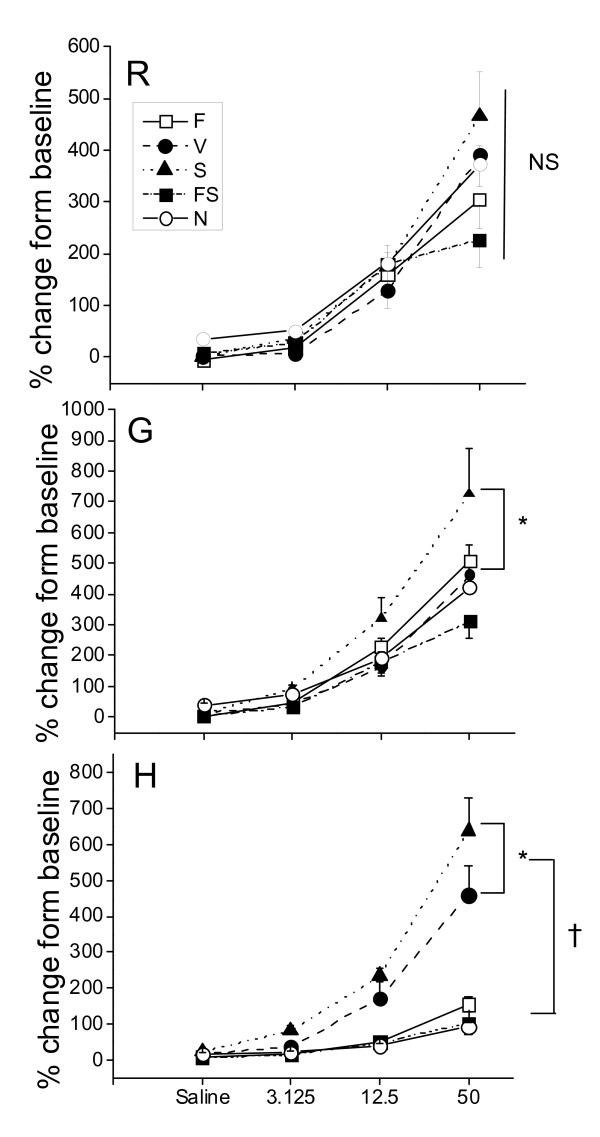
**Lung Mechanics**. Mechanics parameters following nebulized saline and increasing concentrations of methacholine (peak response as percent of baseline). Groups; naïve mice (N) (n = 7) and OVA mice treated with vehicle (V) (n = 6), salmeterol (S) (n = 7), fluticasone (F) (n = 8), salmeterol and fluticasone (FS) (n = 8). R = *R*_*N *_= Newtonian resistance, G = tissue damping, H = tissue elastance. Results expressed as mean ± SE Panel with R: NS no significant differences between the groups. Panel with G: * p < 0.05 for S compared to V, N, F, or FS. Panel with H: * p < 0.05 for S compared to V (p is also < 0.05 for S compared to N, F, or FS). † p < 0.05 for S or V compared to N, F, or FS. All comparisons in this figure are by a two way ANOVA followed by Tukey pairwise comparisons.

Baseline lung mechanics parameters (*R*_*N*_, *G*, and *H*) were essentially equivalent between the treatment groups (see Additional File 2, Figure S2). Overall, the biggest differences between the treatment groups occurred in *H *(Elastance) (see Figure 5). The S group had the greatest change in *H *with a 6-7 fold increase above baseline, with the next biggest response occurring in the V group. Moreover, in both these groups the constant-phase model of lung mechanics was frequently unable to provide a satisfactory fit to impedance at the highest methacholine doses (see Additional File 2, Figure S3). Mice in the N, F and FS groups all had similar responses to methacholine. Salmeterol treatment alone caused a significant increase in *G *(tissue resistance or damping). There was no significant different in *R*_*N *_between any of the groups at any methacholine dose. Mice treated with fluticasone and salmeterol together (FS) generally demonstrated the lowest level of airways hyperresponsiveness in inflamed mice compared to those treated with either salmeterol or fluticasone alone.

### Computed Tomography

Micro-CT images revealed probable atelectasis in distal lung regions in OVA treated mice (See Additional File 2, Figure S4). These findings were not completely eliminated by any of the treatments. Lung volume measured as the thoracic gas volume calculated from the CT (V_TG_) was not significantly different among any of the groups (data not shown).

### Mucin

**Figure 6:**
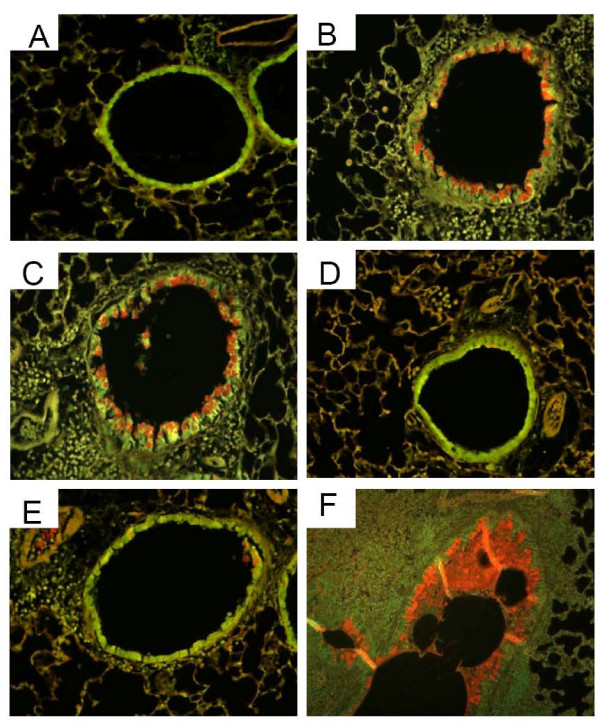
**Mucus Staining**. Representative* PAFS stained tissue sections imaged with a dual excitation filter (FITC/Texas Red) and the 20× objective (F imaged at 10×.). A) naïve, B) OVA, C) Salmeterol, D) Fluticasone, E) Fluticasone and Salmeterol, F) immersion fixed lung from OVA mouse demonstrating airway obstruction with mucus. *Representative figures were chosen using criteria described in the text.

**Figure 7:**
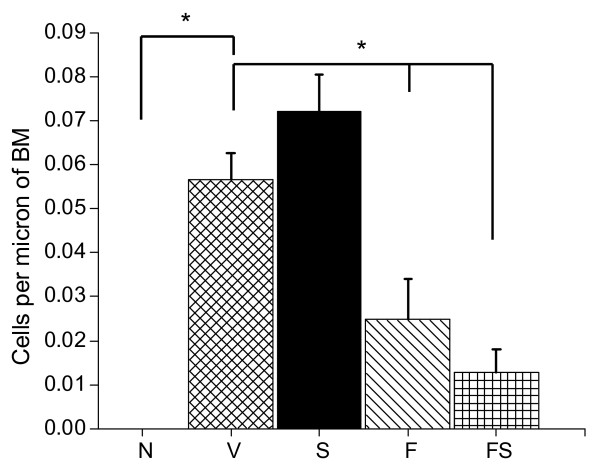
**Mucus Quantification**. Mucus containing (PAFS positive) cells. Groups include naïve mice (N) and OVA sensitized and challenged mice treated with vehicle control (V), salmeterol (S), fluticasone (F), and a combination of fluticasone and salmeterol (FS). Results are expressed as means ± SE. N = 4 mice with 4 airways averaged per mouse (slide). ANOVA p < 0.0001. * p < 0.05.

The number of airway epithelial cells containing airway mucin was greatest in the V and S groups and was significantly less in the F and FS groups (Figures 6 and 7). There was a trend for increased PAFS positive cells in the S group compared to the V (Figure 7) but this did not reach statistical significance.

## Discussion

The goal of the present study was to elucidate if, and to what extent, ICS and LABA, both separately and in combination, alter the pathophysiology of allergic asthma. We found that fluticasone by itself, as might be expected, completely reversed the inflammatory changes assessed both by bronchoalveolar lavage and histologic sections (Figures 2, 3, and 4). Alternatively, treatment given throughout the antigen challenge phase such as ICS may prevent the inflammatory changes from being initiated by having a direct effect on the lung (e.g. innate immunity). In either case, lung remodeling, particularly in terms of mucus metaplasia and epithelial thickening, was essentially abrogated (Figures 4, 6, and 7) and correspondingly, methacholine responsiveness was returned to (or remained at) baseline levels (Figure 5). These findings are in keeping with the well established efficacy of ICS that results from their broad anti-inflammatory activity and that makes ICS the treatment of choice for asthma [7,28].

In stark contrast to the beneficial effects of ICS, treatment with the LABA salmeterol alone increased hyperresponsiveness (Figure 5) assessed at a time point when bronchodilation should be minimal since the measurements were made 24 hours after the last dose of salmeterol and the baseline resistance is not significantly different (Additional File 2, Figure S2). The S group exhibited significantly more total cells than the naive controls and mice treated with fluticasone (Figure 2). While there are statistically insignificant increases in inflammation (Figure 2), epithelial damage (Figure 4), or mucus production (Figure 7), we think that an increase in mucus containing cells or mucus within the airway, in combination with epithelial injury or increased inflammation too subtle to be quantified by simple histological measurements could explain the physiological findings. Alternatively, LABA treatment might have a more direct effect on mucin containing cells that is independent of any effects on the inflammatory response. We base these conclusions on a number of interrelated findings and deductions. First, using computational modeling, we have previously shown that increased airways hyperresponsiveness can be explained by increased epithelial thickening and airway closure [6]. Mucus metaplasia would be expected to enhance airway closure and the S group tended to show increased mucus cell numbers. Consistent with this is the putative role of mucus plugging in fatal human asthma cases [3]. Second, while the trend towards an increase in mucus containing cells within the airway did not reach statistical significance, it is important to point out that the distribution of airway closure is decidedly not uniform [4]. Histological measurements that were done are averaged through the lung and would be expected to lack the sensitivity to detect the changes that are clearly amplified in physiological measurements. Third, the concept that beta agonists may upregulate mucus is supported by previous work implicating beta agonists in mucus production in rats [29], as well as in airway epithelial cell proliferation and airway wall thickening or injury [30]. Fourth we have showed that hyperresponsiveness in elastance (*H)*, a measure of airway closure to methacholine challenge is extremely sensitive to small increases in epithelial thickness and/or airway secretions through the formation of liquid bridges that occlude the lumen of small airways [4,6,21,31,32]. This is supported in the present study by CT imaging that is consistent with airway collapse in the S group. Finally, we found that the constant-phase model frequently did not fit measurements of impedance very well in this particular treatment group, consistent with instability of airway patency and airway closure (See Additional File 2, Figure S3) [21]. Thus, taken together the increased AHR manifested in the parameter H suggests that augmentation in AHR by S is due to enhanced airway closure likely the result of mucus metaplasia and/or epithelial changes.

The current study supports the hypothesis that extended therapy with LABA monotherapy worsens airways hyperresponsiveness, possibly by upregulating either aspects of the inflammatory response or mucin containing cells and exacerbating distal airway closure thus, providing a potential explanation for the rare severe adverse events associated with LABA mono-therapy in asthmatic patients [10,11]. Asthma deaths were first ascribed to the use of beta agonists more than a decade ago [33], and there have been scattered reports that beta agonists can increase secretory cell numbers in human airways [34]. Also, airway closure has been demonstrated to be an important feature in human asthma [35]. It is therefore possible that peripheral airway closure played a role in the LABA-related deaths. Of course, one could argue that the acutely inflamed mice utilized in the present study have limited relevance to the chronic disease of human adult asthma. On the other hand, the FDA recently brought attention to the possible adverse consequences of salmeterol use in the pediatric population [36] which our acute antigen-challenged mouse model may more closely reflect. Although the result of the present study seems to fit with corresponding observations in human asthmatics, they must be viewed in the light of certain limitations. Foremost among these is the fact that mice have important physiological and pharmacologic differences to humans, and that the model of allergic asthma we used reflects simply the acute inflammatory response to a single foreign protein. There may also be differences in the delivery of drugs by nebulization compared with dosing a powdered formulation. Initial titration studies with fluticasone demonstrated evidence of dose dependant anti-inflammatory effects of fluticasone suggesting adequate delivery. We used this model because it has a number of practical advantages, has been well characterized, and exhibits at least some of the features thought to be central to human asthma [6]. And while there are a wide variety of other inflammatory animal models or investigative techniques that exist [37], each of these approaches has its limitations and advantages.

The most important finding of the present study is that the adverse physiological consequences and likely, any related inflammatory or early remodeling changes attributable to salmeterol seem to be completely avoided if LABA is administered in conjunction with fluticasone (Figures 2, 2, 3, 4, 5, 6, 7). This finding is consistent with a recent meta analysis of human clinical data showing the deleterious effects of LABA appear to be abrogated by concomitant use of ICS [38]. Indeed, the combination therapy used in our study was at least as effective as fluticasone alone, and may even have been slightly better when all of the outcomes are taken together. Even so, the anti-inflammatory role of salmeterol remains controversial [15,16,39]. The principal rationale for combination therapies in asthma remains the notion that ICS allow for the benefits of LABAs while at the same time mitigating their disadvantages. In other words, combining these two drugs produces an effect that is not simply the sum of their individual effects. Exactly why synergy should exist between ICS and LABA is not entirely clear. One possibility is beta agonists directly activate the glucocorticoid receptor [9,40]. Alternatively, we have recently demonstrated synergistic interactions between the peripheral remodeling of allergic inflammation and enhanced central airway narrowing in mice [21]. Thus, there is more than one reason why a combination therapy would be superior as one agent treats inflammation and the other treats abnormal smooth muscle function and may involve previously underappreciated mechanisms.

Structural remodeling has long been linked to asthma and this topic has been heavily reviewed [1]. What is unclear is what portion of these structural changes lead to the greatest changes in lung function. Fibrotic changes traditionally considered targets for therapy may in fact; serve a protective role in reducing AHR [2,25]. On the other hand, early changes such as those seen in this model including epithelial thickening and mucus production may produce a more significant decrement in lung function and hyperresponsiveness representing the physiologically important early elements of the remodeling process [41-43]. Several potential therapies impact mucus metaplasia including the MARCKS related peptide that can reduce mucus release into airways [44,45]. Cysteinyl leukotrienes receptor antagonists have been shown to reduce mucus plugging, smooth muscle hyperplasia, and subepithelial fibrosis [46]. Surprisingly, beta blockers have also been shown to reduce mucin content [47]. Taken together with our findings it is reasonable to suggest that airway mucus metaplasia might be a promising therapeutic target in asthma, particularly in patients who are resistant to steroids [28].

## Conclusions

We have investigated the effects of fluticasone and salmeterol, both separately and in combination, on lung structure and function in allergically inflamed mice. Salmeterol alone worsened airways hyperresponsiveness and increased (or failed to reduce) histologic markers of inflammation, remodeling and mucus hyperplasia at least as severely as those associated with untreated inflamed animals. The pattern of hyperresponsiveness was consistent with increased closure of small airways. Concomitant administration of fluticasone maintained or reduced all biomarkers to the level of naüve animals. These results have implications related to the treatment of early asthma and suggest that treatment with LABA alone is detrimental, but that any adverse effects are ameliorated with the combined use of ICS, in support of current clinical practice.

## Abbreviations used

AHR: airways hyperresponsiveness; BALF: bronchoalveolar lavage fluid; BM: basement membrane; COD: coefficient of determination; F: fluticasone; FS: combination of fluticasone and salmeterol; *G*: tissue damping; *H*: tissue elastance; ICS: inhaled corticosteroid; LABA: long acting bronchodilator; O: OVA (ovalbumin); *Rn*: Newtonian Resistance; S: salmeterol; SABA: short acting bronchodilator; V: Vehicle control in addition to OVA; V_TG_: Thoracic gas volume (lung volume calculated from the CT).

## Competing interests

Charles Irvin received support for this research from an investigator-initiated respiratory CRT grant from GSK. Dr. Irvin also reports receiving funding from Merck and Sepracor.

## Authors' contributions

EPR analyzed the data, and performed the histology analysis and wrote the manuscript. MAS modified Image J for histological analysis and reviewed the manuscript, JAT managed the CT scans and assisted with the image reconstruction, HCH assisted with manuscript editing and data analysis, LKL assisted with study design, analysis and manuscript review, JHTB assisted with data review and manuscript preparation, and CGI created the study design, obtained funding and assisted with all data management and manuscript preparation.

All authors have read and approved the final manuscript.

## Supplementary Material

Additional file 1 **Supplemental morphometry methods**. This file contains additional technical information for the morphometry techniques used. Figure S1: This illustrates the quantitative collagen measurement technique using image J software. Click here for file

Additional file 2 **Additional Data including baseline mechanics, z values and CT images**: Figure S2: Baseline lung mechanics parameters (Supplemental Figure 2.doc) Figure S3. Number of z values with a coefficient of determination (COD) less than 0.8. Figure S4: Representative CT images. Click here for file
