# Supplementary material for: Inhaled salmeterol and/or fluticasone alters structure/function in a murine model of allergic airways disease
Source: Respir Res. 2010 Feb 24;11(1):22. doi: 10.1186/1465-9921-11-22 (PMC2841146; doi:10.1186/1465-9921-11-22)
Supplement: Additional file 1 — Supplemental morphometry methods. This file contains additional technical information for the morphometry techniques used. Figure S1: This illustrates the quantitative collagen measurement technique using image J software. [file 1465-9921-11-22-S1.DOC]

**Additional File 1: Supplemental Morphometry Methods**

Semiquantitative morphometric scoring was performed by three observers on randomized masked samples using a Zeiss Axioskop2 plus microscope with an AxioCam and Axiovision software (Carl Zeiss, Gottingen, Germany). Inflammation and epithelial injury were scored on scales of 0-3 on 4 airways per animal. The global epithelial injury score included both thickness and damage to the epithelium. An Olympus BH2 microscope was also used for Sirius red stained sections for polarized imaging with the Zeiss Axiocam and AxioVision software. A FITC-Texas Red filter cube was used to view the red PAFS stain.

Epithelial thickness and mucin containing cells were quantified using Image J software available at [http://rsb.info.nih.gov/ij/download.html](http://rsb.info.nih.gov/ij/download.html.). The program was used to calculate epithelial thickness as the area of the region bounded by the basement membrane and the epithelial border divided by the perimeter length of the basement membrane. Mucin containing cells staining red with PAFS were counted and expressed as number of number of cells per length of basement membrane.

For quantification of peribronchial collagen content, the image J software was modified to analyse polarized photographs from sirius red stained airways. This was done to amplify differences too subtle to be captured with the semiquantitative scoring that revealed no significant differences between groups. Regions of interest were selected frolm color images of each airway, and then anlysis was performed on a polarized gray-scale version of the image. Customized code was added to the program to quantify pixel intesity averaged over a specified region of interest. The region of interest was drawn by a blinded observer choosing a portion of 4 small airways and 4 larger airways per section. Smaller airways were chosen if they had an aspect ration of less than 2:1. Larger airways were sampled in cross section. Airway walls adjacent to blood vessels were excluded. Regions of interest were selected by drawing a line along a segment of the epitherlial border. Image J then extended the region through the basement membrane and into the lung parenchyma to a specified pixel depth (200 pixels for large central airways and 150 pixels for small airways). The line was also extended in the opposite direction into the epithelium by a small distance. Figure S1 (below) shows images and an example of the output from this technique. The pixel intensities were scanned perpendicular to the basement membrane and averaged to produce a plot of mean pixel intensity moving radially outward from the airway lumen. The radial distribution of pixel intensities was then analyzed for its maximum value relative to epithelial baseline, as well as its mean, centroid, skewness, and width at half height.

**Figure S1**: Quantitative collagen measurement technique using Image J software A) Color photo of Sirius Red stained airway border. The region of interest border is shown within the yellow border. B) Grayscale photo of the polarized image of the same section of airway with the corresponding region of interest selected from panel A. C) Average of all the pixels at a given distance from the epithelial border (0) to a position in the lung parenchyma (158).
