# Supplementary material for: Inhaled salmeterol and/or fluticasone alters structure/function in a murine model of allergic airways disease
Source: Respir Res. 2010 Feb 24;11(1):22. doi: 10.1186/1465-9921-11-22 (PMC2841146; doi:10.1186/1465-9921-11-22)
Supplement: Additional file 2 — Additional Data including baseline mechanics, z values and CT images: Figure S2: Baseline lung mechanics parameters (Supplemental Figure 2.doc) Figure S3. Number of z values with a coefficient of determination (COD) less than 0.8. Figure S4: Representative CT images. [file 1465-9921-11-22-S2.DOC]

**Figure S2**: Baseline lung mechanics parameters (*R_N_*, *G*, and *H*). Groups include naïve mice (N), OVAX6 mice treated with vehicle control (V), salmeterol (S), fluticasone (F), and a combination of fluticasone and salmeterol (FS). Results are not significantly different when compared by ANOVA.

**Figure S3**. Number of z values with a coefficient of determination (COD) less than 0.8. The number of z values with a COD less than 0.8 from each set of data taken during a complete methacholine challenge protocol was calculated. Groups include; Naïve mice (N), OVA challenged mice with vehicle control (V), salmeterol (S), fluticasone (F), and a combination of fluticasone and salmeterol (FS). * S is significantly greater than F and FS (ANOVA followed by Tukey’s multiple comparisons test).

**Figure S4**: Representative CT images. The top panel shows isosurface renderings. The bottom images demonstrate axial views of standard CT images. Images are taken from representative animals from the following groups: Naïve mice (N) and OVA treated with vehicle control (V), salmeterol (S), fluticasone (F), and a combination of fluticasone and salmeterol (FS).
